# Effect of shift work on hypertension: cross sectional study

**DOI:** 10.1186/s40557-017-0166-z

**Published:** 2017-04-11

**Authors:** Jeong Han Yeom, Chang Sun Sim, Jiho Lee, Seok Hyeon Yun, Sang Jin Park, Cheol-In Yoo, Joo Hyun Sung

**Affiliations:** Department of Occupational and Environmental Medicine, Ulsan University Hospital, University of Ulsan College of Medicine, 877, Bangeojinsunhwando-ro, Dong-gu, Ulsan, 44033 Republic of Korea

**Keywords:** Shift work, Working period, Hypertension

## Abstract

**Background:**

The need of efficient resource management and full-time accessibility to resources has increased with the development of industry, resulting in the increase of shift workers. Previous researches of past decades show that there are various health effects on shift workers. However, the definition and the form of shift work have varied from each research and occupational harmful factors except for shift work have not been excluded completely in previous researches. Therefore, in this research, we tried to find out the effect of shift work focusing on the hypertension. To complement previously mentioned weakness of other researches, we performed our research on participants to whom we could minimize other risk factors excluding shift work.

**Methods:**

This research examined 1,953 petrochemical plant male workers (shift work 1,075, day worker 878) who did medical checkup from 1st Jan. 2014 to 31th Dec. 2014 in a general hospital located in Ulsan, based on their medical records and questionnaires. With the questionnaire, we found out their basic information including age, social status, occupational history, and we took their physical measurements.

**Results:**

Compared to day workers, shift workers’ odds ratio of developing hypertension was 1.31 (95% CI 0.98–1.75). After adjusting confounding variables, adjusted odds ratio for entire subjects was 1.51 (95% CI 1.11–2.06). Also, for subjects who were in continuous service for over 20 years, odds ratio was 1.51 (95% CI 1.08–2.11).

**Conclusions:**

Shift workers had a higher chance of hypertension than day workers do. Particularly, the longer the workers work continuously, the risk of hypertension getting higher.

## Background

In today’s industrialized world, growing demands for efficient management with use of production facilities and increasing needs for 24-h access to medical services and security have promoted 24-h working environments in modern societies. With increasing number of workers in such industries, the number of shift workers has been consistently rising worldwide [1, 2]. In 2012, 10.9% and 4.9% of male and female workers in South Korea, respectively, were working shifts [3]. In 2010, the European Union reported that 23.0% and 14.0% of male and female workers, respectively, were working shifts, including night shifts [4]. According to the United States labor statistics, 29% workers do the alternative shift and 15% workers are on the regular night shifts in 2010 [5].

The definition of shift work is quite comprehensive. It is usually defined as “work beyond the typical daily working hours (about 7–8 AM to 5–6 PM)”, including graveyard shift, night shift, early morning shift, and rotational work [6–8].

Many reports and research in the past several decades have pointed to the disruption of the endogenous circadian rhythm as the primary effect of shift work on health, which eventually induces the destruction of biological homeostasis [9–11].

Several health effects can be inflicted on shift workers through such a mechanism, of which cardiovascular diseases are one of the most common consequences. In the Korean literature, it has been reported that shift work increases blood pressure, decreases heart rate variability [12], and increases risk for cardiovascular diseases [13, 14]. The studies on other country reported that compared to day workers, shift workers have a higher risk of myocardial infarction, ischemic heart disease, coronary heart disease [15], hypertension, and increased blood pressure [16].

Shift work is also known to increase the risk of gastrointestinal diseases and metabolic disorders. It has been reported that shift workers more frequently experience functional digestive disorders [17] with susceptibility to various gastrointestinal diseases, such as digestive ulcers [18], gastroesophageal reflux [19], and it is associated with elevated Body Mass Index (BMI) [20] and overweight [21], increased blood glucose level, insulin-resistance, and risk of diabetes [22]. Many studies have also suggested that shift work increases the risk of cancer development. Based on a study conducted in 2005 regarding the association between shift work and breast cancer, the International Agency for Research on Cancer of the World Health Organization concluded that shift work has potential carcinogenic effects on humans because of the disruption in the biological circadian rhythm and classified shift work as a probable carcinogen for humans (group 2A) in 2007 [23].

As shown above, many studies have suggested that shift work may cause a variety of adverse health effects, and South Korea authorities have also recognized its seriousness. As a result, the Korea Occupational Safety and Health Agency conducted the “Study of a development of the contents and diagnostic methods of the special health examination for night-shift workers” in 2012 [24]. The Enforcement Rule of the Occupational Safety and Health Act was revised on June 12, 2013 based on the findings of this study. The revision defined night shift as work between 10 PM to 6 AM and stipulated that workplaces with more than 300 full-time workers must provide night shift special health examinations from January 1, 2014 for workers who work a minimum of 4 night shifts per month on an average for 6 months or an average of 60 h or more of night shift per month.

However, the acknowledgement of shift work as a hazard for cardiovascular diseases by the Enforcement Rule of the Occupational Safety and Health Act remains a cause of controversy. Studies in the United States and Japan have shown significant increases of blood pressure caused by shift work [16, 25]. On the other hand, a study also suggested that shift work is not associated with increased blood pressure or hypertension [26].

Furthermore, studies on shift work have applied a wide-ranging definition of shift work, which led to widely varying classifications and forms of shift work in subject selection, where workers performing short-term shift work were included with those perform full-time shift work [6, 8]. Moreover, occupational hazards other than shift work were not completely excluded in some studies [11]. In addition, it was difficult to examine the health effects of long-term shift work because few studies have studied workers who have continuously engaged in shift work during service period [27]. Investigative methods were also not standardized, which has limited past studies from taking a systematic approach to exploring various health effects of shift work [2–4].

The aim of this study was to investigate the effect of shift work on hypertension among various health effects of shift work known so far, for which the standardized body measurements and questionnaires included in the special health examination were utilized.

## Methods

### Study population

**Fig. 1:**
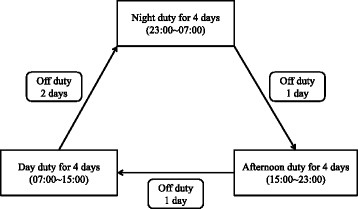
Duty schedule of subjects

This study was conducted using the health examination results and questionnaires of male workers at a petrochemical plant who underwent health exams in a general hospital in Ulsan between January 1, 2014 and December 31, 2014. As the primary duties of most petrochemical plant shift workers are comprised of device manipulations and monitoring which are all performed indoors and field inspections. These workers have little exposure to occupational risk factors other than shift work. In addition, shift workers of a petrochemical plant are placed on a four-crew three-shift system during service period unless there are special circumstances. Figure 1 shows the shift types of the petrochemical plant workers included in this study (Fig. 1).

**Fig. 2:**
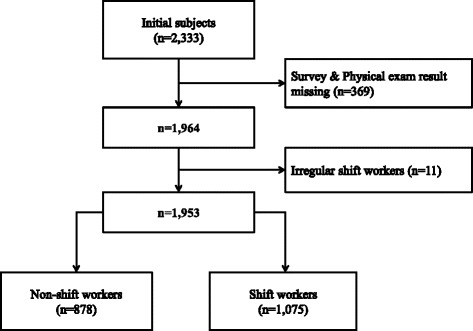
Selection criteria of subjects

From 2,333 potential candidates, a total of 1,953 male adult workers were selected for the final analysis after excluding 369 people for lack of health exam results or health questionnaire data and 11 people for working irregular shifts other than a four-crew three-shift system. Of these subjects, 878 were day workers and 1,075 were shift workers (Fig. 2).

### General characteristics and physical examination

We investigated the subjects’ age, drinking habits, exercise habits, smoking status, and occupational history based on their health exam and questionnaire results. High-risk drinking was defined as a minimum of seven shots on average in one seating with a minimum of two drinking sessions per week [28]. Subjects were considered engaging in adequate exercise if they performed a minimum of 30 min of exercise that induced a mild shortness of breath for at least three times a week [29]. To classify smoking status, we referred to the Standard for Health Examination (Ministry of Health and Welfare Notification No. 2015-19). Subjects who answered that they have never smoked a cigarette or have smoked less than 100 cigarettes throughout their lives were classified into the non-smokers group and subjects who are currently not smoking but had smoked more than 100 cigarettes throughout their lives were classified into the past-smokers group. Subjects who are currently smokers were classified into the current smokers group [30]. Height, weight, systolic and diastolic pressure (SBP and DBP) were measured, and BMI was calculated by dividing weight (kg) by height squared (m^2^).

### Selection and exclusion criteria

We first reviewed the subjects’ health questionnaires taken in the past 3 years (2012-2014) to screen out and classify subjects who were taking hypertension drugs into the hypertension group because drugs can mask the actual blood pressure and hinder us in identifying patients with hypertension. Among subjects who were not taking hypertension drugs, those with SBP ≥ 140 mmHg or DBP ≥ 90 mmHg were classified into the hypertension group [31].

### Statistical analysis

We divided the subjects into the day work and shift work groups to compare their age, height, weight, BMI, SBP, and DBP via Student’s t tests, and subjects’ smoking status, drinking habits, exercise habits, and presence of hypertension via chi square tests. Furthermore, we classified the workers into less than or equal to 10 years, more than 10 years but less than or equal to 20 years, and more than 20 years groups in accordance with their working period and compared the intergroup differences in hypertension prevalence rate and trends via chi square tests and trend analysis. To measure the risk for hypertension, an odds ratio (OR) was calculated via logistic regression based on day work, and an adjusted odds ratio (aOR) was calculated via a multiple logistic regression to adjust for confounding variables (age, BMI, drinking habits, exercise habits, and smoking status). All data were analyzed using IBM SPSS Statistics for Windows, version 21.0 (IBM SPSS Inc., Chicago, IL), and *p*-values less than 0.05 were considered statistically significant.

## Results

A total of 1,953 subjects were included in the final analysis, of which 878 (45.0%) were day workers and 1,075 (55.0%) were shift workers. The mean age of the shift workers (42.5 years) was lower than that of the day workers (43.1 years), but the difference was insignificant.

There was no significant difference in the heights between the two groups, but shift workers’ weight and BMI (73.1 kg and 24.4 kg/m^2^, respectively) were significantly higher than those of day workers (72.1 kg and 24.2 kg/m^2^, respectively) (weight, *p* = 0.011; BMI, *p* = 0.023). In addition, shift workers had significantly higher SBP and DBP (122.4 mmHg and 73.7 mmHg, respectively) than those of day workers (121.0 mmHg and 72.2 mmHg, respectively; SBP, *p* = 0.012; DBP, *p* < 0.001).

A significantly higher proportion of day workers (36.1%) engaged in high-risk drinking compared to shift workers (31.2%, *p* = 0.021), while a significantly higher proportion of shift workers (44.7%) engaged in adequate exercise compared to day workers (31.5%, *p* < 0.001). There were no significant differences in smoking status between the two groups.

In terms of the prevalence rate of hypertension of shift and day workers, 12.2% of the shift workers and 9.6% of day workers had hypertension, but the difference was insignificant (Table 1).

**Table 1:** General characteristics of subjects at baseline

	Non-shift workers (*n* = 878)	Shift workers (*n* = 1,075)	*p* value
Age (years)	43.1 ± 9.3	42.5 ± 7.7	0.099
Height (cm)	172.7 ± 5.3	173.0 ± 5.2	0.182
Weight (kg)	72.1 ± 8.6	73.1 ± 8.7	0.011
Body Mass Index (kg/m^2^)	24.2 ± 2.4	24.4 ± 2.5	0.023
Systolic Blood Pressure (mmHg)	121.0 ± 11.4	122.4 ± 12.0	0.012
Diastolic Blood Pressure (mmHg)	72.2 ± 8.5	73.7 ± 9.1	<0.001
High risk alcohol drink^a^			
No	561 (63.9)	740 (68.8)	0.021
Yes	317 (36.1)	335 (31.2)	
Adequate exercise^b^			
No	601 (68.5)	595 (55.3)	<0.001
Yes	277 (31.5)	480 (44.7)	
Smoking status			
Non-smoker	248 (28.2)	271 (25.2)	0.132
Ex-smoker	317 (36.1)	433 (40.3)	
Current smoker	313 (35.6)	371 (34.5)	
Hypertension			
No	794 (90.4)	944 (87.8)	0.066
Yes	84 (9.6)	131 (12.2)	

Unit: mean ± standard deviation, number (percentage)

*p*-value was calculated by Student *t* test for continuous variables

*p*-value was calculated by Chi-square test for categorical variables

^a^High risk alcohol drink: drink more than 2 times per week and more than 7 glasses per one time

^b^Adequate exercise: exercise harder than daily activity for more than 30 min and exercise more than 3 times per week

The hypertension rate of all subjects significantly increased with increasing years of working, where the rate was 4.6% for workers with less than or equal 10 years of working, 9.5% for workers with more than 10 but less than or equal to 20 years of working, and 16.4% for workers with more than 20 years of working (*p* < 0.001). The trend analysis showed significant differences in accordance with the working period (*p* < 0.001). For day workers, prevalence of hypertension in workers with less than or equal 10 years of working was 5.0%, which increased to 6.8% in workers with more than 10 but less than or equal to 20 years of working, 15.1% in workers with more than 20 years of working, and the differences were significant (*p* < 0.001). The trend analysis showed significant differences as well (*p* < 0.001). For shift workers, the prevalence of hypertension in workers with less than or equal 10 years of working was 4.1%, which increased to 10.8% in workers with more than 10 but less than or equal to 20 years of working and again increased to 17.3% in workers with more than 20 years of working, and the differences were significant (*p* < 0.001). The trend analysis showed significant differences as well (*p* < 0.001) (Table 2).

**Table 2:** The prevalence rate of hypertension according to working period

		0 ~ 10 years	10.1 ~ 20 years	more than 20 years	*p* value for trend
Total	No	599 (95.4)	408 (90.5)	731 (83.6)	<0.001
(*n* = 1,953)	Yes	29 (4.6)	43 (9.5)	143 (16.4)	
Non-shift workers	No	343 (95.0)	136 (93.2)	315 (84.9)	<0.001
(*n* = 878)	Yes	18 (5.0)	10 (6.8)	56 (15.1)	
Shift workers	No	256 (95.9)	272 (89.2)	416 (82.7)	<0.001
(*n* = 1,075)	Yes	11 (4.1)	33 (10.8)	87 (17.3)	

Unit: number (percentage)

With day workers as the baseline, the OR of hypertension in all shift workers was 1.31 (95% CI 0.98–1.75). When shift workers were stratified in accordance with working period, the OR of hypertension in workers with less than or equal 10 years of working was 0.41 (95% CI 0.21–0.78), and that for workers with more than 20 years of working was 1.98 (95% CI 1.43–2.73). The aOR after adjusting for variables that may affect blood pressure (age, BMI, drinking habit, exercise habit, and smoking status) for all shift workers was 1.51 (95% CI 1.11–2.06) and that for workers with more than 20 years of working was 1.51 (95% CI 1.08–2.11) (Table 3).

**Table 3:** Odds ratios and adjusted odds ratio of hypertension according to working period

Variables	Crude OR^a^ (95% CI)	Adjusted OR^b^ (95% CI)
Work schedule		
Day work	1.00	1.00
Shift work	1.31 (0.98-1.75)	1.51 (1.11-2.06)
Work duration (years)		
0 (day work)	1.00	1.00
≤ 10	0.41 (0.21-0.78)	1.68 (0.73-3.89)
10.1 – 20	1.15 (0.75-1.76)	1.48 (0.92-2.39)
> 20	1.98 (1.43-2.73)	1.51 (1.08-2.11)

^a^Odds ratio was calculated by logistic regression analysis

^b^Adjusted odds ratio was calculated by multiple logistic regression analysis after adjusting for age, BMI, alcohol drinking, exercise and smoking

## Discussion

This study compared the prevalence rate of hypertension between day workers and shift workers on a four-crew three-shift system at a petrochemical plant in South Korea. There are two main results. First, the prevalence of hypertension showed an increasing tendency with shift work duration. Second, workers engaged with shift work more than 20 years had a significant risk for hypertension.

Past studies have suggested that compared to day workers, shift workers are more vulnerable to metabolic disorders, such as diabetes and metabolic syndrome [22], and cardiovascular disorders, such as hypertension [32], due to a disruption of circadian rhythm caused by irregular working schedules. Such diverse health effects are induced by a common mechanism via circadian rhythm disruption. Melatonin, which is secreted by the pineal gland under the regulation of the suprachiasmatic nucleus in the hypothalamus, is known to be the first hormone that regulates the circadian rhythm [33]. The secretion and inhibition of melatonin is affected by the presence of light, and melatonin is known to regulate the diurnal gene of the gonads and peripheral tissues via the hypothalamus-pituitary gland-gonad axis and hypothalamus-pituitary gland-adrenal gland axis and also to play a role in blood pressure control by acting on vascular endothelial cells [34]. According to recent studies, melatonin released by the pineal gland binds to the melatonin 2 receptor of vascular endothelial cells to activate the L-arginine pathway, which increases the production of nitric oxide and stimulates the production of soluble guanylate cyclase within the vascular smooth muscle, increasing cyclic guanosine monophosphate and ultimately being involved in vascular relaxation [35]. Hence, normal melatonin secretion is intimately associated with blood pressure control. However, blood pressure control would be more difficult in shift workers than in day workers, because their irregular light exposure, which is associated with melatonin secretion, would disrupt the circadian rhythm. As predicted, when we compared all subjects, shift workers had significantly higher risks for hypertension than day workers in this study.

Furthermore, this study also verified that shift workers with more than 20 years of working had significantly higher risk for hypertension than day workers before and after adjusting confounding variables. An existing Korean study reported significantly increasing blood pressure with an increasing shift-work period [12], but the results denoted only short-term health effects as the average shift-work period in the previous study was only 5.2 years (5.4 months–10 years). Nevertheless, our findings suggest that who do the shift work longer, have more clear association with hypertension compared to relatively short term shift worker.

In conclusion, we observed that the risk for hypertension was higher in shift workers than in day workers and this finding is consistent with results reported in previous studies [32]. In particular, we observed that long-term shift work increases the risk for hypertension. Alternatively, shift workers with a short to medium-term working period did not show a significant increase in the risk of hypertension. Past studies on the association between shift work and cardiovascular diseases have suggested that risk for coronary artery disease increases in workers with more than 5 years of shift work [8]. Studies on hypertension suggested that blood pressure increases with the increasing period of shift work [12, 13, 16]. However, no study has identified the specific working period for shift workers after which the risk for hypertension clearly increases.

In the present study, risk for hypertension did not significantly increase in workers with less than 5 years of working and those with more than 5 but less than or equal to 21 years of working, but there is an important note to consider. A statistics report based on The Fifth Korea National Health and Nutrition Examination Survey (KNHANES V-1) a hypertension prevalence rate in Korean men in 2010 of 32.4% [28], which is quite different from the 9.6% (day workers) and 12.2% (shift workers) found in the present study. This may be a result of the possible exclusion of individuals with hypertension or with a high risk for hypertension during hiring due to the healthy worker effect [36]. Therefore, it is difficult to estimate the period at which risk for hypertension clearly increases solely based on the findings of our study, but our results still indicate that shift workers should pay closer attention to blood pressure control by regularly checking their blood pressure, adjusting lifestyle habits, and striving to reduce the length of shift work.

This study has a few limitations. First, this study did control confounding variables, but as a cross-sectional study, it cannot draw clear conclusions with regard to causal relationships. Second, unhealthy people or people with diseases are not hired or they retire early, hence, the prevalence rate of disease may have been underestimated due to the healthy worker effect, which makes the existing workers seem healthier than in reality [36]. Third, among the participants, some exceptional shift worker changed their work schedule as day work, but we couldn’t count on the exact number nor excluded them in the analysis. Fourth, the types of shift work in the present study circulated in a reverse direction, where workers moved from night shift to afternoon shift and morning shift, and therefore we could not examine the health effects associated with various types of shift work. Fifth, we did not have information on possible confounders such as socioeconomic status, which may be related weakly to hypertension. Finally, the questionnaire used in this study was a form developed for special health examinations and the subjects were screened out depending on the criteria for a special health examination. Hence, we could not conduct a questionnaire survey on all subjects and could not use additional questionnaires.

Despite these limitations, this study has several strengths. First, since researches on shift work can include various types of shift work, it is difficult to conduct a study only on shift workers on an identical shift system. However, this study eliminated differences depending on shift work patterns by only including shift workers on a four-crew three-shift system. So we can find out the association between hypertension and this type of shift work. Second, the subjects of past studies on shift workers were usually subway workers, car factory workers, and nurses based on National Health and Nutrition Examination Survey. In these studies, it is difficult to distinguish the effects of shift work typically, since many occupational factors other than the shift work itself could affect blood pressure. Furthermore, these workers may not be continuously in shift work throughout their career at the workplace because of duty change, change jobs or department. In this study, the health effects of shift work are well reflected in the examined shift workers because they were operators at a petrochemical plant who engaged in shift work from the first day to the last day of work unless there were special circumstances. Moreover, their main duties comprised indoor tasks (operating, monitoring) and inspection that did not pose a serious health risk (these workers particularly have relatively little exposure to hypertensive hazards). Third, this study utilized the special health exam results of shift workers as defined by the Occupational Safety and Health Act, and unless the Act is revised in the future, it will allow standardized tests to be performed for shift workers in the future. Thus, a cohort could be established based on the findings of this study, and long-term follow-up would enable researchers to conduct a prospective study on the health effects of shift work.

## Conclusions

This study analyzed the health effects of shift work and found that shift workers had heightened risks for hypertension compared to the day workers, and the increase of risk was especially marked in workers with longer working periods.

Hence, establishing national and social measures to protect the health of shift workers and provide a safer and improved working environment for shift workers will contribute to enhancing the quality of life of shift workers, ultimately curtailing social and financial costs.

## Abbreviations

aOR: Adjusted odds ratio
BMI: Body mass index
DBP: Diastolic blood pressure
OR: Odds ratio
SBP: Systolic blood pressure
